# Arterial Spin-labeling in Central Nervous System Infection

**DOI:** 10.2463/mrms.mp.2015-0140

**Published:** 2016-03-21

**Authors:** Tomoyuki NOGUCHI, Yusuke YAKUSHIJI, Masashi NISHIHARA, Osamu TOGAO, Koji YAMASHITA, Kazufumi KIKUCHI, Muneaki MATSUO, Shinya AZAMA, Hiroyuki IRIE

**Affiliations:** 1 Department of Radiology, National Center for Global Health and Medicine, 1–21–1 Toyama, Shinjuku-ku, Tokyo 162-8655, Japan; 2 Department of Radiology, Saga University; 3 Department of Neurology, Saga University; 4 Department of Clinical Radiology, Kyushu University; 5 Department of Pediatrics, Saga University

**Keywords:** arterial spin-labeling, brain perfusion, central nervous system infection, HSV encephalitis, meningitis

## Abstract

**Purpose:**

To investigate the characteristics of arterial spin-labeling magnetic resonance imaging (ASL-MRI) in central nervous system (CNS) infection.

**Methods:**

Thirty-two patients with CNS infections underwent a pulsed ASL-MRI. The findings on ASL-MRI were retrospectively assessed for the pathogens as well as each of the following four pathology classified based on conventional MRI findings: non-purulent parenchymal involvement, meningeal involvement, abscess formation, and ventricular involvement.

**Results:**

Among the 17 patients with non-purulent parenchymal involvement, ASL-MRI revealed high perfusion in 8 patients (47%) and low perfusion 1 patient (6%). Especially, four of five patients (80%) with definite or suspected herpes simplex virus (HSV) infection showed high perfusion on ASL-MRI. Seventeen of 22 patients (77%) with meningeal involvement showed high perfusion along the cerebral sulci irrespective of the pathogens. Meanwhile, 4 of 16 lesions (25%) with abscess formation showed low perfusion and one of six patients (17%) with ventricular involvement had high perfusion.

**Conclusions:**

The characteristics of ASL-MRI in CNS infections were clearly delineated. ASL-MRI could be helpful for monitoring the brain function in CNS infections noninvasively.

## Introduction

Central nervous system (CNS) infection is a serious condition that induces inflammation of the leptomeninges or brain parenchyma. It carries a risk of severe sequelae as well as a poor prognosis, and thus early diagnosis and treatment are important.^1,2^

In patients with suspected CNS infection, neuroimaging is performed to diagnose the condition, to differentiate it from other brain disorders, and to examine the features and distribution of lesions. Magnetic resonance imaging (MRI) is particularly helpful in providing detailed information about the disease. However, MRI findings depend considerably on various factors, such as the type of the causative microorganism (virus, bacteria, fungus, or parasite), the age and/or condition of patient (newborn, elderly, immunosuppressed, and so on), and possibly even on prior antibiotherapy,^1,2^ and the images are sometimes unclear despite clinically pronounced manifestations. Therefore, further development of neuroimaging techniques is required to overcome the diagnostic difficulty.

In recent years, arterial spin-labeling (ASL) has been developed as a new type of MRI (ASL-MRI). ASL-MRI can measure brain perfusion information in 3 to 5 minutes without the infusion of extrinsic tracers. Because of its noninvasive nature, ASL-MRI can be used to safely obtain cerebral blood flow images even in children and pregnant women. ASL-MRI has demonstrated the clinical efficacy in cases of brain tumor,^3^ dementia,^4^ stroke,^5^ dural arteriovenous fistula,^6^ moyamoya disease,^7,8^ and so on. Although characteristic findings of brain perfusion imaging have been reported for CNS infection, most of the studies employed nuclear-medicine- or contrast-enhanced-MRI-based perfusion imagings, while only a few reports employed ASL-MRI.^9,10^ In addition, differences in the relevance of abnormal findings between ASL-MRI and other conventional MRI remain undefined. Therefore, the purpose of this study was to retrospectively investigate the characteristics of ASL-MRI in CNS infections as well as its limitations.

## Materials and Methods

This retrospective research was approved by our institutional review board (IRB), and the requirement for informed consents was waived by the IRB (No. 2013-09-01).

### Patients

Patients with suspected CNS infections who underwent MRI including ASL-MRI in the period from June 2008 to September 2013, were identified using the radiology information system (RIS) at our hospital. Then the hospital information system (HIS) was employed to select only those patients who met the following diagnostic criteria: (a) manifestation of neurological symptoms, (b) abnormal findings on MRI, and (c) detection of pathogenic microorganism or abnormal findings in cerebrospinal fluid (CSF) including pleocytosis (5 or more/μL) or elevated protein level (more than 40 mg/dL). We excluded patients with Creutzfeldt-Jakob disease, autoimmune limbic encephalitis, multiple infarctions or ruptured infected aneurysms due to infectious endocarditis, or infectious meningoencephalitis secondary to craniotomy procedure. Patients without abnormal findings on MRI were excluded even if they were diagnosed based on the detection of pathogens, because in this study abnormal findings on MRI were used to identify the location of CNS lesions.

### Magnetic resonance imaging

ASL-MRI and other MRI sequences were carried out as part of the routine clinical brain MR examinations performed on a clinical 1.5-Tesla MRI unit (MAGNETOM Avanto; Siemens AG, Erlangen, Germany) with an 8-channel head coil or a 3.0-Tesla MRI unit (MAGNETOM Trio A Tim System; Siemens AG) with a 12-channel head coil.

#### 1. ASL-MRI

ASL-MRI images were obtained using the sequence called quantitative imaging of perfusion with thin-slice TI1 periodic saturation, second version (Q2TIPS), a pulsed ASL-MRI method that enables the acquisition of multiple sections.^11^ Subsequently, cerebral blood flow (CBF) mapping (ASL map) was computed voxel-wise based on the ASL-MRI images according to the following equation proposed by Wang et al.^12^:

(1) f=λ×δM/(2α×M0×TI1×exp(−TI2/T1a))

where *f* is the regional cerebral blood flow, λ is the brain/blood partition coefficient of water, δM is the difference in the longitudinal magnetization between the unlabeled and labeled images in the region of interest (ROI), α is the inversion efficiency, M0 is the equilibrium magnetization of the regional brain tissue, and T1a is the longitudinal relaxation time of arterial blood. TI1 is the given waiting time after an inversion recovery radiofrequency (IR) pulse for labeling arterial blood in a labeling slab. TI2 is the given time between the IR labeling pulse and imaging data acquisition. In the present study, the parameters were as follows: TI1/TI2 (ms) = 700/2000; labeling slab width/imaging slab width/gap between the two slabs (mm) = 100/114/25; λ (g/mL) = 0.9; α = 0.95; T1a (ms) = 1496.19 at 3.0-Tesla and 1200 at 1.5-Tesla; imaging sequence = single-shot echo-planar imaging (EPI) gradient-echo sequence; repetition time (TR)/echo time (TE) (ms) = 2500/17; field of view (mm) = 256 × 256 × 114; slice thickness/interslice gap (mm) = 6/1.2; the number of slices = 16; coverage area = whole brain; slice acquisition order = sequentially proximal-to-distal; matrix = 64 × 64; voxel size (mm) = 4 × 4 × 6; phase partial Fourier ratio = 7/8; flow limit (flow velocity cutoff value for crusher gradients that eliminate fast-moving spins) (cm/s) = 4; and the number of acquired image volumes ranged from 41 to 91 (first image volume acquired without a labeling pulse as an M0 image volume, and 20 to 45 pairs of labeled and unlabeled image volumes) resulting in an acquisition time (minutes: seconds) of 2: 04 to 3: 57 for Q2TIPS.

#### 2. Other MR imaging sequences

For most patients enrolled in this study, MRI was performed before a definitive diagnosis was established; therefore various kinds of MRI sequences were applied to each patient. For comparison with ASL-MRI, the following imaging conditions were basically used for a referential standard as follows: non- or postcontrast fluid-attenuated inversion recovery (FLAIR) images, postcontrast T_1_-weighted images (T_1_WIs), or diffusion weighted images (DWIs).

FLAIR was performed using a turbo-spin-echo-based inversion recovery sequence with the following parameters: TR/TE/inversion time (TI) (ms) = 9000/83 – 99/2500; FOV (mm) ranged from 114 × 200 to 182 × 220; inversion flip angle (degrees) = 150–170; slice thickness /interslice gap (mm) = 5/1 or 6/1.2; matrix size ranged from 179 × 256 to 242 × 384; pixel spacing (mm) ranged from 0.6 × 0.6 to 0.9 × 0.9; number of slices = 20–22. If the administration of intravenous gadolinium (Gd) contrast agent was performed, the acquisition of precontrast FLAIR was skipped and only postcontrast FLAIR was obtained.

DWI was acquired using a single-shot EPI diffusion-weighted spin-echo sequence with one additional image acquired without diffusion-weighting (b value = 0 s/mm^2^). The parameters were as follows: Number of diffusion-weighting directions (number of directions = 3 and 12; b value = 1000 s/mm^2^ ; TR/TE (ms) = 4000/90 and 5200 − 5800/91.0 − 94.2; FOV (mm) ranged from 200 × 150 to 220 × 220; flip angle (degrees) = 90 and 90; slice thickness /interslice gap (mm) = 6/1.2 and 4/0; matrix size ranged from 128 × 96 to 128 × 128; pixel spacing (mm) ranged from 1.6 × 1.6 to 1.7 × 1.7; number of slices = 20–40. Postcontrast T_1_WI was obtained after the administration of Gd contrast agent with the volume interpolated breath-hold examination (VIBE) sequence with the following parameters: TR/TE (ms) = 8.54 – 15/3.26–4.04; FOV (mm) ranged from 165 × 220 to 186 × 220; flip angle (degrees) = 10–12; slice thickness / interslice gap (mm) = 0.8–0.9 / 0; matrix size ranged from 192 × 256 to 234 × 288; pixel spacing (mm) ranged from 0.8 × 0.8 to 0.9 × 0.9; number of slices = 160–240.

The intravenous injection of Gd contrast agent was performed according to the physician’s request depending on the patient’s condition. The agents used were 0.1 mmol/kg gadolinium with diethylenetriamine pentaacetic acid (Gd-DTPA) (Magnevist; Bayer HealthCare Japan, Osaka), Gd-DTPA-BMA (Omniscan; Daiichi Sankyo Co., Ltd., Tokyo), or Gd-HP-DO3A (Prohance; Eisai Co., Ltd., Tokyo).

### Visual assessment methods

MR images were evaluated independently by three observers who were blind to the medical records of the patients. All three (KK, with 3 years of experience in neuroradiology; KY, with 6 years of experience in neuroradiology; OT, with 9 years of experience in neuroradiology) worked at another hospital apart from the hospital where patients were enrolled in the current study. They observed all images on a computer viewer system (ViewR version 1.09.15; Yokogawa Electric Corporation, Tokyo) with a 54-cm class color LCD monitor (Radioforce R22; Eizo Nanao, Ishikawa).

### ASL-MRI evaluation

Patients showed a variety of abnormal findings on conventional MRIs. Abnormal findings from each patient were subdivided into four pathological categories—non-purulent parenchymal involvement, meningeal involvement, abscess formation, and ventricular involvement—based on the findings on postcontrast T_1_WI, non- or postcontrast FLAIR, or DWI. One estimator (TN, with 9 years of experience in neuroradiology) reviewed the conventional MRI and subdivided the abnormal findings.

Non-purulent parenchymal involvement was determined by the existence of an abnormal high intensity area in the parenchyma on FLAIR. Meningeal involvement was determined by the existence of an abnormal high intensity area along the surface of the parenchyma on FLAIR. Abscess formation was defined as the ring-like enhancement on postcontrast T_1_WI as well as the high intensity within the lesion on DWI. Ventricular involvement was diagnosed by the existence of an abnormal high intensity area on FLAIR or linear enhancement on postcontrast T_1_WI in the periventricular regions. Concurrent intraventricular empyema and choroiditis were also estimated as optional findings. Empyema was defined as high intensity fluid collection within the ventricles on DWI. Choroiditis was determined by abnormal enlarged choroidal lesions with enhancement on T_1_WI. These four pathologies might overlap one another. That is, non-purulent parenchymal involvement could be found with other pathological conditions including meningeal involvement or ventricular involvement. Abscess formation could also exist with non-purulent parenchymal involvement if there was an absolutely normal parenchymal area between them.

ASL maps generated by ASL-MRI were used for visual assessment. All ASL maps were displayed by using the color lookup table processed by a workstation (AZE virtual place Raijin, AZE, Ltd., Tokyo).

As an evaluation step, the observers were first provided ASL images alone, and then they were provided with pathological information if the lesions were not detected. The diagnostic confidence of ASL-MRI was rated according to the following four-point scale, and then the findings on ASL-MRI were determined as low or high perfusion compared to the surroundings in case of the lesions which were estimated as moderate, sufficient, or excellent.

1. Unclear delineation (normal perfusion)
2. Moderate delineation (abnormal perfusion is detected in comparison with referential standard of FLAIR, DWI, or postcontrast T_1_WI)
3. Sufficient delineation (abnormal perfusion is detected when informed of the pathological category)
4. Excellent delineation (abnormal perfusion is definitely detected without any additional information)

Cohen’s kappa coefficient of the diagnostic confidence of ASL-MRI for each of the four pathological categories was measured to evaluate the inter-observer agreement of this grading system among the three observers.^13^ Landis’ judgment standard was adopted to determine the level: values >0.8 were considered to suggest almost perfect agreement, values >0.6 showed substantial agreement, values >0.4 showed moderate agreement, values >0.2 showed fair agreement, and values >0 showed negligible agreement.^14^

When two or all three of the observers agreed on a grade, the concordant value was adopted as the final grade. Otherwise, the median value among them was adopted as the final grade. Then, the detection rates on ASL-MRI were estimated for each of the four pathological categories.

## Results

### Patient population

Table 1 shows the characteristics and findings for each patient. Thirty-two patients (19 males and 13 females; age range 3–86 years; median 53.5 years) were enrolled in this study.

The following pathogens were identified in 14 patients: herpes simplex virus (HSV) (n = 3), rotavirus (n = 1), a combination of John Cunningham virus (JCV) and human immunodeficiency virus (HIV) (n = 1), tuberculosis bacterium (n = 3), nocardia (n = 2), streptococcus (n = 1), and cryptococcus (n = 3). Another 13 patients with unspecified viral infection and 5 with unspecified bacterial infection were clinically diagnosed based on clinical symptoms, cerebrospinal fluid pleocytosis, and elevated cerebrospinal protein levels. Patients presented initially with one or several overlapping neurological symptoms including abnormal behavior (n = 1), epilepsy (n = 6), headache (n = 12), physical weakness (n = 5), impaired consciousness (n = 12), general fatigue (n = 1), higher brain dysfunction (n = 2), and appetite loss (n = 1). Seven patients had an underlying illness, including acute myelocytic leukemia treated by bone-marrow transplantation (n = 1), adult T-cell leukemia virus (ATLV) infection (n = 2), multiple myeloma (n = 1), whole body contusion (n = 1), alcoholic liver disease (n = 1), and concurrent systemic lupus erythematosus, diabetes mellitus, and bronchopneumonia (n = 1).

### ASL-MRI evaluation

**Fig. 1.**
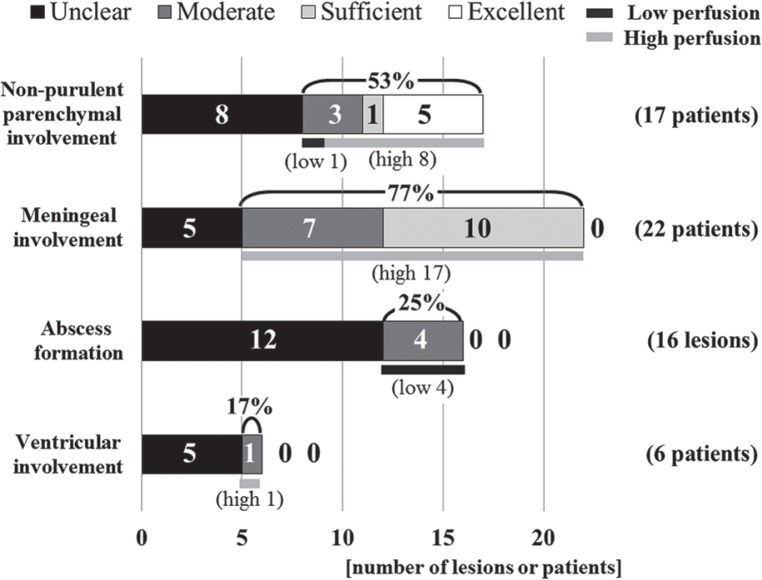
Results of the diagnostic confidence for ASL-MRI. Among the 17 patients with non-purulent parenchymal involvement, 9 patients (53%) showed abnormal perfusion on ASL-MRI. High perfusion on ASL-MRI was demonstrated in 17 of 22 patients (77%) with meningeal involvement. Meanwhile, ASL-MRI revealed abscess formation and ventricular involvement in low rates (25% and 17%, respectively). ASL-MRI, arterial spin-labeling magnetic resonance imaging.

Figure 1 shows the final grading values resulting from the evaluation of the diagnostic confidence and the finding of ASL-MRI. The interobserver agreements on the diagnostic confidence of ASL-MRI in non-purulent parenchymal involvement, meningeal involvement, abscess formation, and ventricular involvement were judged to be substantial, moderate, substantial, or almost perfect, respectively (Cohen’s kappa coefficient = 0.75, 0.41, 0.56, and 0.89, respectively).

**Fig. 2.**
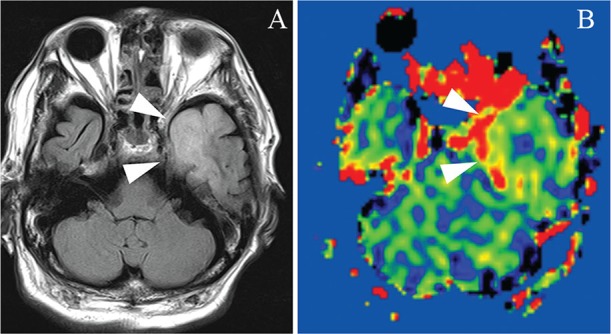
A 78-year-old man with non-purulent parenchymal involvement due to HSV infection. Fluid-attenuated inversion recovery shows a high intensity area in the left medial temporal lobe, which is typical in HSV encephalitis (**A**: arrowheads). The high perfusion in that area is pronounced on arterial spin-labeling magnetic resonance imaging (**B**: arrowheads). HSV, herpes simplex virus.

Of the 17 patients with non-purulent parenchymal involvement, high perfusion was detected in eight patients (47%) (Fig. 2). Especially, two of three patients with HSV infection showed high perfusion on ASL-MRI. In addition, two patients with an unspecified viral infection (No. 6 and No. 7) who showed high perfusion in the affected medial temporal lobe were clinically suspected of having HSV encephalitis, but the pathogen could not be identified. That is, a high incidence (80%, 4/5) of high perfusion on ASL-MRI could be observed in patients with definite or suspected HSV encephalitis. On the other hand, in one patient (8%) with a combination of JCV and HIV, low perfusion was detected on ASL-MRI in areas where high intensity was observed on FLAIR.

**Fig. 3.**
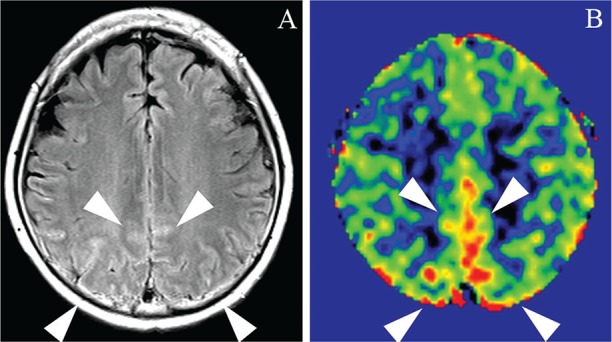
A 63-year-old woman with meningeal involvement due to unspecified viral infection. High intensity along the cerebral sulci is observed on fluid-attenuated inversion recovery (**A**: arrowheads). High perfusion is prominent in the affected leptomeningeal areas on arterial spin-labeling magnetic resonance imaging (**B**: arrowheads).

Of the 22 patients determined to have meningeal involvement, high perfusion on ASL-MRI was seen in a high percentage of patients (77%, 17/22) irrespective of pathogens (Fig. 3).

**Fig. 4.**
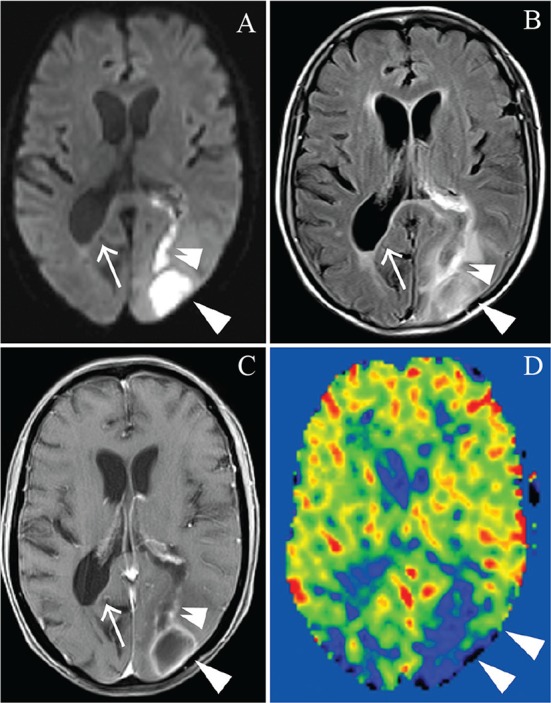
An 80-year-old woman with abscess formation and ventricular empyema due to unspecified bacterial infection. Diffusion weighted image and fluid-attenuated inversion recovery depict the abscess formation (**A**, **B**: arrowheads) with intraventricular penetration (**A**, **B**: double arrowheads). In addition, these images delineate the development of ventricular inflammation as a high intensity area along the posterior wall of the right lateral ventricle (**A**, **B**: arrows). Postcontrast T_1_WI (weighted image) shows ring-like enhancement around the abscess (**C**: arrowheads) as well as enhancement along the perforating path (**C**: arrow) and right posterior periventricular wall (**C**: double arrowheads). Arterial spin-labeling magnetic resonance imaging shows low perfusion only around the abscess areas (**D**: arrowheads), and reveals no abnormal perfusion in the ventricular wall.

A total of 16 abscess lesions were observed in five patients. Four lesions (25%) of three patients were detected as low perfusion on ASL-MRI (Fig. 4) and the other 12 lesions were indistinctive.

**Fig. 5.**
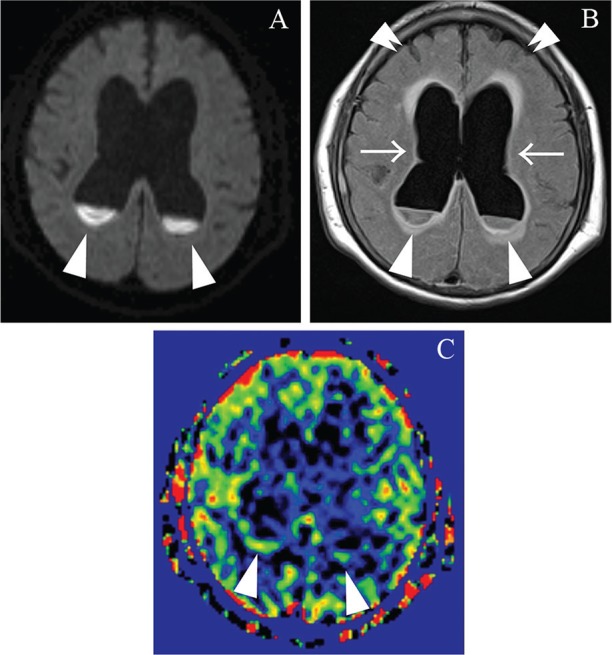
An 85-year-old woman with ventricular involvement due to unspecified bacterial infection. Diffusion weighted imaging and FLAIR show the purulent fluid collections as high intensity areas pooled in the posterior horns of the bilateral lateral ventricles (**A**, **B**: arrowheads). In addition, FLAIR demonstrates not only periventricular inflammation as periventricular high intensity areas in the ventricular walls, but also hydrocephalus as dilated ventricles (**B**: arrows) in conjunction with compacted sulci of the bilateral cerebral hemispheres (**B**: double arrowheads). Arterial spin-labeling magnetic resonance imaging shows bracket-like high perfusion along the posterior walls of the bilateral lateral ventricles (**C**: arrowheads). FLAIR, fluid-attenuated inversion recovery.

Six patients were determined to have ventricular involvement including a ventricular empyema in five patients and choroiditis in one. All six patients had other overlapping lesions, including abscess formation in three, non-purulent parenchymal involvement in one, and meningeal involvement in five patients. This result might suggest that these ventricular lesions represented a concomitant condition secondary to the other regional infections. ASL-MRI revealed only one of the six patients (17%) with ventricular involvement of empyema due to unspecified bacterial infection as high perfusion along the ventricular wall where abnormal enhancement was observed on postcontrast T_1_WI (Fig. 5).

## Discussion

CBF examinations for CNS infection have traditionally been accomplished through perfusion imaging based on nuclear medicine studies, dynamic contrast-enhanced computed tomography (CT), or dynamic susceptibility contrast MRI (DSC-MRI). For example, hypoperfusion of the whole brain was reported in HIV encephalopathy.^15^ On the other hand, patients with Japanese encephalitis were shown to exhibit abnormal CBF increases in the affected thalami and putamina.^16^ Interestingly, patients with HSV encephalitis were reported to show increased blood flow in the medial temporal lobe when technetium with hexamethylpropyleneamine oxime (^99m^Tc-HMPAO) was used, but not when technetium with ethyl cysteinate dimer (^99m^Tc-ECD) was used, which suggested the presence of tracer-specific dynamics in CBF examinations.^17^ However, nuclear medicine examinations cannot be performed frequently in clinical practice because they require special facilities, expensive agents, and invasive examination methods. Dynamic CT and MRI perfusion imaging also have disadvantages, including the need to administer contrast material and procedural difficulties such as with the bolus injection technique.^8^

ASL-MRI is one of the non-contrast-enhanced MR perfusion imaging methods. ASL-MRI has several advantages, such as noninvasiveness, no additional costs, and lack of complicated procedures. To date, two studies have been published on the use of ASL-MRI in patients with CNS infection.^9,10^ Ances et al. reported that resting CBF within the lenticular nuclei and visual cortex in patients with HIV infection was reduced prior to neuropsychological impairment.^9^ Khoury et al. reported that the presence of hyperperfusion was inversely related to the occurrence of immune reconstitution inflammatory syndrome at the time of scan.^10^ However, their results have limited clinical application, and a more comprehensive review will be needed.

Our study was the first investigation which broadly enrolled patients to generally survey the utility of ASL-MRI in the diagnosis and evaluation of CNS infection. Among the non-purulent parenchymal involvement group, two of three patients with HSV infection showed high perfusion on ASL-MRI. In addition, two patients with high perfusion due to unspecified viral infection were both clinically suspected of HSV encephalitis without firm evidence. While the perfusion findings were different between ^99m^Tc-HMPAO and ^99m^Tc-ECD, as mentioned above,^17^ our study followed ^99m^Tc-HMPAO perfusion imaging. The findings on ASL-MRI may have corresponded to the severe inflammatory reaction observed in acute fulminant hemorrhagic necrosis of HSV encephalitis,^18^ or can be caused by epileptic activities, both clinical and subclinical. ASL-MRI would be useful to differentiate HSV encephalitis from other T_2_-prolonged neoplastic lesions such as diffuse astrocytoma.

Approximately 77% of patients with meningeal involvement demonstrated high perfusion along the affected meninges detected on FLAIR or postcontrast T_1_WI. No investigation has been performed to examine the perfusion in meningitis. ASL-MRI can visualize arterial blood flow feeding both intra- and extra-axial regions, as long as the flow passes through the labeling area that is placed in the cervical region.^6^ High perfusion on ASL-MRI might visualize hyperemia in the meningeal lesions or represent the epileptic activities as mentioned above. Although FLAIR is highly sensitive for meningitis,^19^ meningeal hyperintensity on FLAIR is not uncommon in other differential diseases involving the leptomeninges,^20^ such as subarachnoid hemorrhage^21^ and meningeal carcinomatosis.^22^ ASL-MRI, in combination with FLAIR, might be helpful in differentiating these diseases.

On the other hand, 4 of 16 lesions in six patients with abscess formation expressed low perfusion in the abscess areas on ASL-MRI. Muccio et al. reported a series of patients with brain abscess formation with low relative cerebral blood volume (rCBV) measurements by DSC-MRI.^23^ They explained that the capsule of pyogenic abscesses was prevalently composed of collagen fibers with a low capillary density and reduced rCBV. This pathological finding may also apply to some abscess lesions with low perfusion on ASL-MRI. In clinical use, ASL-MRI might be helpful in differentiating abscess formation from a rare glioblastoma containing a hyperintense cystic lesion on DWI,^24^ because glioblastoma generally demonstrates ring-like high perfusion^3^ but abscess formation shows unclear or low perfusion in our results.

A low rate of visualization on ASL-MRI was found in patients with ventricle involvement (17%). In fact, all six patients with ventricle involvement had other concurrent lesions. This result may suggest that the ventricular lesions in our study were secondary to some other regional infection. Thus, it was possible that the ventriculitis arose through the spread of inflammation from other lesions, and that the relatively immature vascular formation resulted in inconclusive ASL-MRI results. Only one patient showed bracket-like high perfusion along the lateral ventricular walls in the bilateral posterior horn. ASL-MRI might express microvessel development in the affected lesions. Anyhow, there has been no perfusion study that investigated ventricular lesions.

This study had several limitations. First, the number of subjects was small. However, it would be enough to speculate the trends of ASL-MRI in CNS infection. On the basis of our initial results, further investigations with larger numbers of subjects will be planned to confirm the clinical application of ASL-MRI to CNS infection. Another limitation was that histopathological confirmation was not obtained for either the ASL-MRI findings or the conventional MRI findings. However, histopathological confirmation by open biopsy or postmortem examination may be practically difficult. It will be necessary to accumulate evidence by performing various less-invasive examinations to improve the reliability of our current findings. The other limitation is that the current ASL-MRI findings may have been affected by motion artifacts. Further improvement of ASL-MRI will be needed to obtain higher quality perfusion images.

In summary, ASL-MRI showed reasonably good and consistent image quality even in patients with CNS infection. About a half of non-purulent parenchymal lesions were detected in various perfusion pattern. Seventy-seven percent of meningeal involvement demonstrated high perfusion along the leptomeninges. A fourth of abscess lesions showed low perfusion. Only 17% of ventricular involvement had high perfusion along the ventricular wall. ASL-MRI was thus effective at characterizing CNS infection as functional perfusion abnormalities.

## Figures and Tables

**Table 1.** Patient list

Patient number: Age/Sex	Pathogen	Neurological symptoms	Period between CSF examination and MRI (day)	Tesla	Administration of gadolinium contrast agent	ASL findings^*^			

						Non-purulent parenchymal involvement	Meningeal involvement	Abscess formation	Ventricular involvement
1: 78/M	HSV	abnormal behavior, epilepsy	4	3.0	no	high	unclear		
2: 53/M	HSV	epilepsy	6	3.0	yes	high			
3: 73/M	HSV	physical weakness, impaired consciousness	10	3.0	no	unclear			
4: 3/M	rotavirus	impaired consciousness	0	1.5	no	high			
5: 38/M	JCV and HIV	impaired consciousness	0	1.5	yes	low			
6: 76/M	unspecified virus	general fatigue, appetite loss	4	3.0	no	high			
7: 58/M	unspecified virus	impaired consciousness	0	3.0	no	high			
8: 23/M	unspecified virus	headache, physical weakness	4	3.0	yes	unclear			
9: 36/M	unspecified virus	epilepsy	0	3.0	no	high	high		
10: 29/M	unspecified virus	headache	0	1.5	no	high	high		
11: 41/M	unspecified virus	epilepsy	9	1.5	no		high		
12: 63/F	unspecified virus	headache	1	3.0	yes		high		
13: 28/F	unspecified virus	epilepsy	1	3.0	no		high		
14: 80/F	unspecified virus	impaired consciousness	3	3.0	yes		high		unclear
15: 20/M	unspecified virus	epilepsy	0	1.5	yes		high		
16: 15/M	unspecified virus	headache	5	3.0	yes	unclear	unclear		
17: 54/M	unspecified virus	headache, impaired consciousness	0	1.5	yes		unclear		
18: 30/F	unspecified virus	higher brain dysfunction	0	3.0	yes		high		
19: 48/M	tuberculosis bacterium	headache, physical weakness, impaired consciousness	1	1.5	yes	unclear	high		
20: 41/F	tuberculosis bacterium	headache	10	3.0	yes		high		
21: 44/F	tuberculosis bacterium	headache, impaired consciousness	13	3.0	no	unclear		unclear (2 lesions)	unclear
22: 73/M	nocardia	physical weakness	19	3.0	no			unclear (6 lesions)	
23: 73/F	nocardia	physical weakness	1	3.0				low	
24: 18/M	staphylococcus	headache	1	3.0	yes	unclear	high		
25: 52/M	streptococcus	impaired consciousness	0	3.0	yes		high	unclear (4 lesions) and low (2 lesions)	unclear
26: 61/F	cryptococcus	headache	1	1.5	yes	unclear	high		
27: 78/F	cryptococcus	impaired consciousness	4	3.0	yes		high		
28: 73/F	cryptococcus	physical weakness, higher brain dysfunction	7	1.5	yes		unclear		unclear
29: 80/F	unspecified bacterium	headache	2	1.5	yes		unclear	low	unclear
30: 85/F	unspecified bacterium	impaired consciousness	1	1.5			high		high
31: 75/M	unspecified bacterium	headache	2	1.5	yes	high	high		
32: 86/F	unspecified bacterium	impaired consciousness	0	1.5	no	unclear	high		

*: The blanks in the “ASL findings column” indicate that no abnormality was observed on conventional MRIs. ASL, arterial spin-labeling; CSF, cerebrospinal fluid; F, female; JCV, John Cunningham virus; HSV, herpes simplex virus; M, male; MRI, magnetic resonance imaging.
